# Clocking the Lyme Spirochete

**DOI:** 10.1371/journal.pone.0001633

**Published:** 2008-02-20

**Authors:** Stephen E. Malawista, Anne de Boisfleury Chevance

**Affiliations:** 1 Department of Internal Medicine, Yale University School of Medicine, New Haven, Connecticut, United States of America; 2 Centre d'Ecologie Cellulaire, Hôpital de la Salpétrière, Paris, France; Massachusetts General Hospital, United States of America

## Abstract

In order to clear the body of infecting spirochetes, phagocytic cells must be able to get hold of them. In real-time phase-contrast videomicroscopy we were able to measure the speed of *Borrelia burgdorferi (Bb),* the Lyme spirochete, moving back and forth across a platelet to which it was tethered. Its mean crossing speed was 1,636 µm/min (N = 28), maximum, 2800 µm/min (N = 3). This is the fastest speed recorded for a spirochete, and upward of two orders of magnitude above the speed of a human neutrophil, the fastest cell in the body. This alacrity and its interpretation, in an organism with bidirectional motor capacity, may well contribute to difficulties in spirochete clearance by the host.

## Introduction

*Borrelia burgdorferi (Bb)* locomote by the beating of two opposing sets of endoflagella which alternatively drive them in opposite directions along their axes. They swim using backward propagating flat waves, much like the waves found in eukaryotic cells such as sperm [1]. Their speed in liquid media has been estimated at 4.25 µm/sec, or 255 µm/min [1]. They are much faster (up to ∼2000 µm/min) in gel-like viscous media such as methylcellulose (simulating tissue ground substance) [2] or on surfaces, where they are thought to have something to push against [3], [4].

## Results and Materials and Methods

Bb are known to engage activated platelets via alpha IIb beta 3 integrin receptors [5]. For phagocytic studies [6] we took platelet-rich plasma from (sodium) heparinized human blood, added a low passage clonal isolate of B. burgdorferi strain N40 cultivated as described [7], sealed the suspension with paraffin between a glass slide and cover slip, and examined the preparation on the warmed (33°C) stage of a Zeiss phase-contrast photomicroscope connected via a IEC800 Microscope Video Camera (elc, Annecy, France) to a Panasonic Time Lapse Video Recorder AG6720 (Matsushita Electric Industrial Co., Osaka). Platelets adhered to the slide; many spirochetes, to the platelets.

We noted that spirochetes tethered to platelets are easier to ingest, as they cannot leave the field as free spirochetes can. In still photographs of these negotiations we noted that with time, different regions along the length of the spirochete are in contact with the platelet. In time-lapse videomicrocopy (Video S1) *Bb* were moving back and forth across the surface of the platelet, sometimes changing direction during translocation, but stopping short on reaching either end and remaining there until the reverse propulsion kicked in (the control of these reversals is unknown). Then they moved back across the platelet in the opposite direction. These translocations could happen repeatedly.

**Figure 1:**
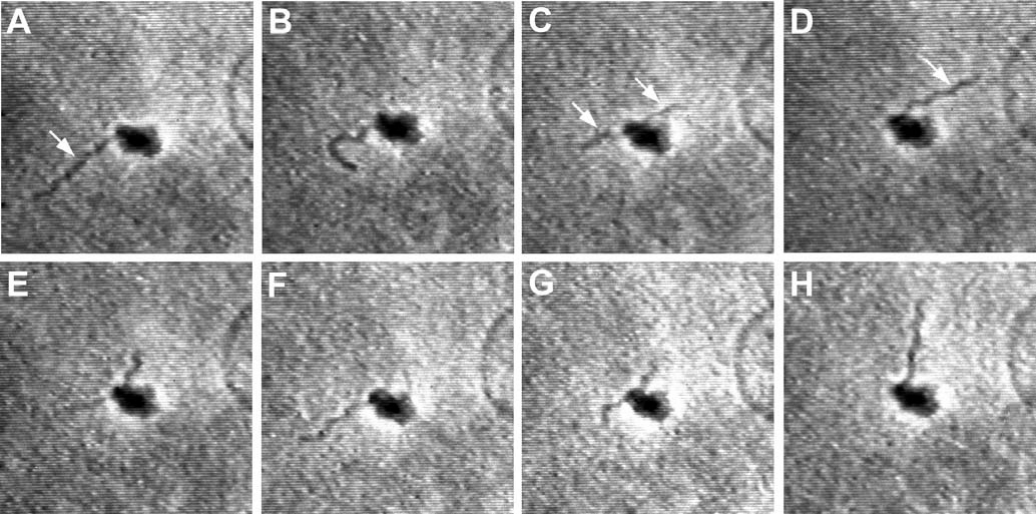
Sequential translocations of Bb across a platelet (see text). Total elapsed time, A-H: 9 sec. Measured crossing speeds were as fast as 2800 µm/min, upward of two orders of magni-tude above the speed of a human neutrophil, the fastest cell in the body. Images from real-time phase-contrast videomicroscopy. Approx. ×1,000.

Examples filmed in real time are seen in Figure 1. In this preparation the tethered spirochete made repeated complete crossings from one of its ends to the other. This made its speed amenable to analysis. We examined frame by frame the time it took for the *Bb* to cross the platelet in either direction, and, having crossed, how long it spent at either extremity. We made these measurements for 14 crossings (7 in each direction) over 34 sec, and, a little later, another 14 crossings over 42 sec. Combining the two sets, we calculated a mean crossing time of 0.55 sec (S.D.±0.19 sec), compared to a mean time between crossings of 1.71 sec (S.D.±1.23). The difference between these means is highly significant (n = 28; P<0.0001, paired t test, two tailed). Moreover, the spirochete measured 15 µm in length; therefore, its mean crossing speed was ∼27 µm/sec, or 1,636 µm/min. Its fastest crossing, measured in three of the 28 crossings, was 0.32 seconds, giving a fastest crossing speed of 46.88 µm/sec, or ∼2800 µm/min, which we believe to be the most rapid spirochete speeds so far measured.

## Discussion

There is good previous evidence that antigens can move longitudinally with some facility along spirochetal membranes–from antibody-coated latex beads attached to the spirochete, *Leptospira interrogans*–but their estimated speeds were only up to 660 µm/min [8]. All these speeds must seem blindingly fast to neutrophils, which crawl (they do not swim) at ∼20 µm/min [9], [10] in liquid media. The disparity in speed is likely to be even more marked in the extravascular space, as spirochetes move easily in gel-like viscous media [2] but neutrophils average only ∼4 µm/min [11]. The most likely explanation for what we are seeing is that the ligand-receptor complexes, as in *Leptospira* [8], are freely movable along the spirochete's outer membrane sheath. Receptors on Bb appear well distributed about their surface but may become concentrated in patches when given antibody [12]. We suspect that these translocations can occur rapidly. If, instead of its receptors moving, *Bb* were rapidly exchanging receptors as it moved along the platelet surface, we might expect it to fly off the platelet when it reached its end. The fact that *Bb* stop short at each of their ends is compatible with its accumulating and dragging ligand-receptor complexes to the *Bb*'s end, where they constrain the spirochete from departing. In addition to their alacrity and bidirectional motor capabilities, if movable receptors on spirochetes prove to be a general phenomenon, then strong ligation, or locomotor disabling, would also seem to be required if it is to be internalized efficiently by phagocytes.

## Supporting Information

Video S1 *Bb* moving back and forth along a platelet. Time-lapse (16x normal) phase-contrast videomicrocopy.
(10.30 MB MOV)
